# The *RFC1 80G>A*, among Common One-Carbon Polymorphisms, Relates to Survival Rate According to DNA Global Methylation in Primary Liver Cancers

**DOI:** 10.1371/journal.pone.0167534

**Published:** 2016-12-09

**Authors:** Sara Moruzzi, Silvia Udali, Andrea Ruzzenente, Alfredo Guglielmi, Patrizia Guarini, Nicola Martinelli, Simone Conci, Filippo Mazzi, Patrizia Pattini, Stephanie A. Tammen, Oliviero Olivieri, Francesca Pizzolo, Sang-Woon Choi, Simonetta Friso

**Affiliations:** 1 Department of Medicine, University of Verona School of Medicine, Verona, Italy; 2 Department of Surgery, University of Verona School of Medicine, Verona, Italy; 3 Tufts University School of Nutrition Science and Policy, Boston, Massachusetts, United States of America; 4 Chaum Life Center, CHA University, Seoul, Korea; Universita degli Studi di Napoli Federico II, ITALY

## Abstract

Polymorphisms within one-carbon metabolism genes have been largely studied in relation to cancer risk for the function of this pathway in nucleotide synthesis and DNA methylation. Aims of this study were to explore the possible link among several common functional gene polymorphisms within one-carbon metabolism and survival rate in primary liver cancers, *i*.*e*., hepatocellular carcinoma and cholangiocarcinoma, and to assess the additional effect of global DNA methylation on survival rate and mortality risk. Forty-seven primary liver cancer patients were genotyped for ten polymorphisms: *DHFR 19bp ins/del*, *TS 2rpt-3rpt*, *MTHFD1 1958G>A*, *MTHFR 677C>T*, *MTR 2756A>G*, *MTRR 66A>G*, *RFC1 80G>A*, *SHMT1 1420C>T*, *BHMT 716 A>G*, *TC II 776C>G*. Methylation was determined in peripheral blood mononuclear cells (PBMCs) DNA as methylcytosine (mCyt) content using LC/MS/MS. Among the polymorphisms analysed, the *RFC1 80G>A* (rs1051266) influenced the survival rate in primary liver cancers. The *RFC1 80AA* was associated to a significantly reduced survival rate (22.2%) as compared to both GG and GA genotypes (61.5% and 76% respectively, p = 0.005). When the cancer patients were stratified according to the mCyt median value as high (>5.34%) or low (≤5.34%), the concomitant presence of AA genotype and low mCyt level led to a significantly worse survival rate as compared to the G allele carriership (p<0.0001) with a higher Hazard Ratio (HR = 6.62, p = 0.001). The subjects carrying the AA genotype in association with high mCyt did not show a significant difference in survival rate as compared with the G allele carriers (p = 0.919). The *RFC1 80G>A* polymorphism influenced the survival rate, and the presence of *RFC1 80AA* genotype with low global methylation in PBMCs DNA was associated with poorer prognosis and higher mortality risk, therefore highlighting novel molecular signatures potentially helpful to define prognostic markers for primary liver cancers.

## Introduction

One-carbon metabolism is essential for several intracellular reactions including those involved in nucleotide synthesis and biological methylation, in particular methylation of DNA, the major epigenetic mechanism in mammalian cells [1].

Genetic variants of one-carbon pathway genes have been investigated mainly in colon cancer as potential markers of cancer susceptibility [2–5] for the involvement of this metabolism in cellular development, proliferation and differentiation [6, 7]. The molecular mechanisms underlying the possible association among polymorphic variants of one-carbon metabolism genes, cancer survival rate and mortality risk are, however, not completely clarified.

DNA methylation is a heritable and reversible phenomenon that consists in the covalent binding of a methyl group to the 5’carbon of a cytosine in CpG dinucleotide sequences and it plays a role in gene expression regulation and maintenance of genomic stability [8–10]. An aberrant DNA methylation, both global and gene-specific, is involved throughout all the phases of cancer development and progression, and a status of global DNA hypomethylation is an almost universal finding in cancer tissues [11, 12]. A global hypomethylation in peripheral blood mononuclear cells (PBMCs) DNA was associated to an increased cancer risk [13] and a shorter survival rate in patients affected by different types of cancer [14–16]. In recent studies, we observed significantly reduced methylcytosine (mCyt) levels in PBMCs DNA of cancer patients [17, 18] and lower levels were directly associated to an unfavourable prognosis [18].

As for the possible relationship among one-carbon metabolism gene variants, global DNA methylation and cancer, we previously reported that the TT genotype of the *MTHFR 677C>T* polymorphism associated with low plasma folate levels presented a decreased DNA methylation [19] and higher incidence of cancer [17]. Global DNA hypomethylation in PBMCs in association with the carriership of *MTHFR* T allele represents, therefore, a predicting factor of cancer development and an unfavourable prognostic factor in cancer disease [17]. The potential association among other polymorphic variants of one-carbon metabolism genes, global DNA methylation in PBMCs and survival rate in cancer is still poorly investigated as it is the role of aberrant DNA methylation in clinical outcome and prognosis in cancer disease.

Cancer is a major public health issue [20] and among the different cancer types, primary liver cancers, *i*.*e*. hepatocellular carcinoma and cholangiocarcinoma, are prevailing malignancies with a high worldwide mortality rate [21].

Aims of the present study were: i) to explore the possible link among the most common variants of one carbon-related genes and survival rate in primary liver cancers, *i*.*e*. hepatocellular carcinoma and cholangiocarcinoma, and ii) to assess the additional effect of global DNA methylation on survival rate and mortality risk.

## Materials and Methods

### Study subjects and survival data collection

The study was approved by the Institutional Review Board Ethical Committee of the University of Verona School of Medicine Hospital (Verona, Italy). Written informed consent was obtained from each patient after a detailed explanation of the study.

Forty-seven patients affected by primary liver cancers, 31 hepatocellular carcinoma and 16 cholangiocarcinoma, were enrolled from April 2009 to March 2013 among those referring to the Division of Surgery of the Verona University Hospital for curative surgery intervention. Inclusion criteria were age ≥18 years with the following surgical resectability criteria: preserved liver function, class A Child-Pugh score, absence of extrahepatic metastases. Exclusion criteria were a coexisting human immunodeficiency (HIV), hepatitis B (HBV) or hepatitis C (HCV) viruses infection; presence of relevant concurrent medical conditions such as chronic inflammatory diseases or haematological disorders, including autoimmune liver diseases and hereditary hemochromatosis; presence of an acute inflammatory disease, decompensate liver cirrhosis (Child-Pugh B, C). A trained physician recorded a detailed clinical history data including lifestyle habits. All subjects under B vitamins supplementation and/or using drugs known to interfere with folate-related one-carbon metabolism in the month before enrolment were excluded.

A periodic evaluation of the patients consisting in a complete medical examination or a telephone interview was performed during a follow-up period of 60 months. The follow-up period was calculated from the date of the surgical intervention up to the date of death or to the latest recorded medical examination.

### Biochemical analyses

Samples of venous blood were drawn from each subject after an overnight fasting and analysed by routine laboratory test analysis for a complete blood count and determination of serum C-reactive protein (CRP), creatinine, aspartate transaminase (AST), alanine transaminase (ALT), gamma-glutamyltranspeptidase (gGT), alkaline phosphatase (ALP), total bilirubin, albumin, glycemic level, total cholesterol, triglycerides, prothrombin time-international ratio (PT-INR), ferritin, serological tests for hepatitis B and C viruses. Plasma folate and vitamin B12 were measured by an automated chemiluminescence method (ChironDiagnostics, East Walpole, MA, USA) and total plasma homocysteine concentrations was determined by high-performance liquid chromatography (HPLC) with fluorescent detection [22].

### Genotyping

From each subject venous blood was drawn into Vacutainer^®^ tubes containing EDTA as anticoagulant after an overnight fast and DNA was extracted from PBMCs by Wizard Genomic DNA Purification Kit (Promega Corporation, Fitchburg, WI, USA). One carbon metabolism gene variants were analysed by different methods, as follows: *DHFR* 19bp ins/del [23] and *TS 2rpt-3rpt* [24] by PCR; *MTHFD1 1958G>A* (rs2236225) [25], *MTHFR 677C>T* (rs1801133) [26], *MTR 2756A>G* (rs12749581) [27], *MTRR 66A>G* (rs1801394) [28], *RFC1 80G>A* (rs1051266) [29] and *SHMT1 1420C>T* (rs1979277) [30] by PCR followed by restriction fragment length polymorphism assays, *BHMT 716 A>G* (rs3733890) and *TC II 776C>G* (rs1801198) by allelic discrimination Real Time-PCR technology using the assay C_11646606_20 and the assay C_325467_10, respectively (ABI Prism 7500, Applied Biosystems, Carlsbad, CA, USA).

### Global DNA methylation

Global DNA methylation was determined using a liquid chromatography/mass spectrometry (LC/MS/MS) method and mCyt levels expressed as percent (%)mCyt = [(mCyt)/(mCyt + Cyt)] x 100, as previously described [19, 31], with slight modifications [18]. Briefly, global DNA was extracted from PBMCs and hydrolyzed to nucleosides using 2 units of nuclease P1, 0.002 units of venom phosphodiesterase I and 0.5 units of alkaline phosphatase. Isotope-labelled internal standards for deoxycytidine and 5-methyl-deoxycytidine were added to samples before the run in a 3200 Q Trap MS-MS system coupled with an Agilent 1100 Series liquid chromatograph (Agilent, Santa Clara, CA, USA).

### Statistical analysis

All the statistical computations were performed by using the IBM SPSS Statistics software version 22 for Windows (IBM Inc., Armonk, NY, USA). Continuous variables were expressed as mean values ± standard deviations (SD), parameters showing a skewed distribution were log-transformed and thus expressed as geometric means with 95% confidence intervals (CIs). Continuous variables were tested by analysis of variance (ANOVA) with Tukey's post-hoc comparison when appropriate. All genotype distributions were verified to be in agreement with Hardy-Weinberg equilibrium.

Survival rate was analysed by Kaplan-Meier curves with Log Rank test and pairwise comparison when indicated. Hazard ratio (HR) of mortality with 95% CI was estimated by Cox regression analysis adjusted for age and gender, and by including folate plasma concentration in the regression model. A p-value <0.05 was considered statistically significant.

## Results

### Clinical and biochemical characteristics of primary liver cancer patients

Table 1 reports the main clinical and biochemical characteristics of the 47 primary liver cancer patients (31 hepatocellularcarcinoma, 16 cholangiocarcinoma). The main biochemical analyses were within the normal range and, in particular, the indexes of hepatic function confirmed a compensated liver function status in all patients (Table 1). Viral serologic tests for HBV and HCV were negative in all patients according to the enrolment criteria. As for neoplastic markers, alpha-fetoprotein values were within the normal range (<7 g/l) in 39% of the HCC patients, whereas normal levels of CA 19.9 (<25 U/ml) and CEA (<5 ng/ml) were observed in 50% and 100% of CC patients, respectively.

**Table 1:** Clinical and biochemical characteristics of primary liver cancer patients.

	References values	Cancer patients (n = 47)
**Clinical characteristics**		
Age, years		67.1 ± 9.0
Gender, % male		76.6%
Smoking		68.1%
Alcohol drinking		72.3%
**Laboratory tests**		
CRP (mg/L)*	<5	7.70 (5.13–11.55)
Hb (g/dL)	13.5–16.0	13.5 ± 1.62
MCV (fL)	86–98	91.9 ± 7.49
WBCs (10^9^/L)	4.3–10.0	7.01 ± 2.96
PLTs (10^9^/L)	150–400	235.6 ± 119.9
AST (U/L)*	8–50	41.7 (32.8–53.2)
ALT (U/L)*	8–45	40.8 (30.2–55.0)
ALP (U/L)*	30–130	92.4 (77.9–109.6)
gGT (U/L)*	<50	76.7 (58.6–100.3)
Total bilirubin (mg/dL)*	0.11–1.05	0.71 (0.59–0.86)
Direct bilirubin (mg/dL)*	<0.35	0.25 (0.20–0.31)
PT (INR)*	0.82–1.14	1.11 (1.07–1.15)
Albumin (g/L)	35–50	39.9 ± 5.54
CHE (U/L)	4650–14400	6515 ± 1890
Total cholesterol (mg/dL)	<200	159.5 ± 48.9
Triglycerides (mg/dL)*	<150	112.7 (100.1–126.7)
Creatinine (mg/dL)*	0.59–1.29	0.86 (0.74–1.00)
Glucose (mmol/L)	3.5–5.5	6.39 ± 1.81
Folate (nmol/L)*	10.4–42.4	8.52 (9.95–10.45)
tHcy (μg/L)*	< 15	13.2 (10.4–16.7)
Vitamin B12 (pmol/L)*	142–724	337.0 (288.4–393.8)
Vitamin B6 (nmol/L)*	25–128	19.7 (15.4–25.2)
Ferritin (μg/L)*	30–400	249.4 (178.2–349.0)

Values are expressed as mean ± SD.

*: log-transformed variables are shown as geometric mean with 95% confidence interval

Alcohol drinking defined as ≥ 36 g ethanol/day for males and ≥ 24 g ethanol/day for females

Abbreviations: CRP, C-reactive protein; Hb, Hemoglobin; MCV, Mean Corpuscular Volume; WBCs, White Blood Cells; PLTs, Platelets; AST, aspartate aminotransferase; ALT, aspartate alanine aminotransferase; ALP, Alkaline phosphatase; gGT, gamma glutamyl transferase; PT (INR), Prothrombin International Ratio; CHE, cholinesterase; tHcy, Homocysteine.

### Survival rate according to one-carbon polymorphic variants

The polymorphic variants distribution of all evaluated one-carbon metabolism genes was in agreement with the Hardy-Weinberg equilibrium in the study subjects.

**Fig 1:**
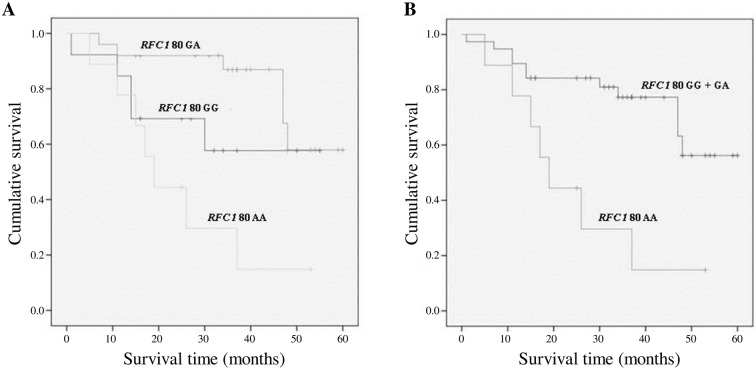
Survival curves plotted by Kaplan-Meier analysis according to *RFC1 80G>A* genotypes. (A) The survival rate was worse in *RFC1 80AA* (22.2%) patients as compared to the *RFC1 80GA* (76%) and *RFC1 80GG* (61.5%) genotypes (p = 0.005). (B) The survival rate was lower among the *RFC1 80 AA* patients as compared with the G allele carriers (*RFC1 80GG*+*GA*) (p = 0.002). The percentage of survivors was 22.2% and 71.1%, respectively.

The Kaplan-Meier analysis to test the survival rate was performed in accordance to the ten gene polymorphic variants object of the study. Among the analysed polymorphisms, only the *RFC1 80G>A* polymorphism influenced the survival rate in primary liver cancers. The RFC1 80G>A genotypes frequencies are reported in Table 2. The homozygous variant *RFC1 80AA* was associated to a lower survival rate (22.2%) as compared to both *RFC1 80 GG* and *GA* genotypes (survival rate 61.5% and 76% respectively, p = 0.005) (Fig 1A). The statistical significance increased when the *RFC1 80AA* subjects were compared to the G allele carriers (*RFC1 80 GG + GA*) (Fig 1B) with a survival rate of 22.2% *versus* 71.1%, respectively (p = 0.002). When the main biochemical parameters, in particular plasma folate levels, were analysed according to the *RFC1 80G>A* variants, no significant differences were observed among the three genotypes except for platelet count, higher in the AA group, and triglycerides concentration that was lower in the GG genotype group (data not shown).

**Table 2:** RFC1 80G>A genotypes frequencies in primary liver cancer patients (n = 47).

	*RFC1 80GG*	*RFC1 80GA*	*RFC1 80AA*
**Number of patients**	13	25	9
**Percentage of patients**	27.7%	53.2%	19.1%

### *RFC1 80G>A* genotypes and survival rate according to DNA global methylation

The observed association of *RFC1 80G>A* polymorphism with survival rate in primary liver cancer (Fig 1) suggested to evaluate mCyt levels according to *RFC1 80G>A* genotypes. The comparison of mCyt levels showed no statistically significant differences among the genotypes (AA 5.41%, GA 5.36% and GG 5.25%, p = 0.607). The methylation status did not differ also when comparing 80AA subjects with G allele carriers (*RFC1 80GG* + *GA*) (5.41% *versus* 5.32%, respectively, p = 0.553) (data not shown).

**Fig 2:**
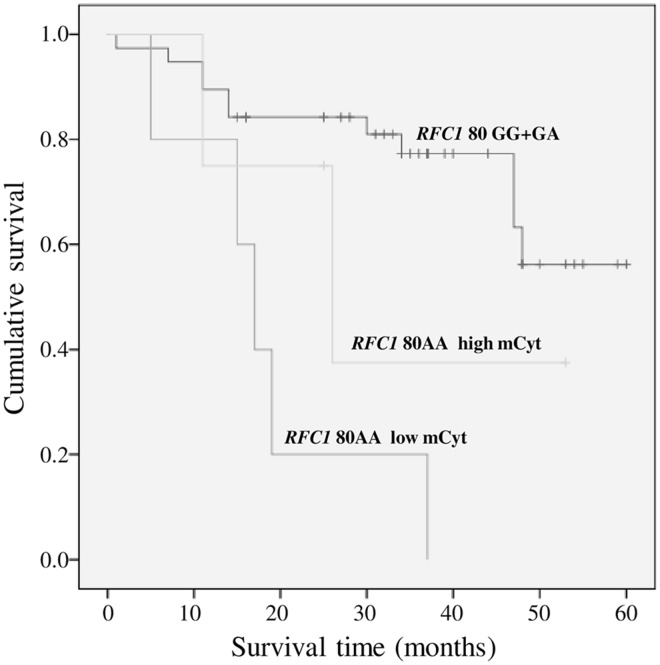
Survival curves plotted by Kaplan-Meier analysis according to *RFC1 80G>A* genotypes and mCyt levels. *RFC1 80AA* patients with low mCyt levels (≤5.34%) were associated to a lower survival as compared to carriers of the *RFC1 80G* allele (71.1%) (p<0.0001). The comparison between *RFC1 80G* carriers and *RFC1 80AA* with high mCyt (>5.34%) was not statistically significant (p = 0.919), as it was the comparison between *RFC1 80AA* with low mCyt and with high mCyt (p = 0.209).

Subsequently the study group was stratified in two subsets according to high (>5.34%) or low (≤5.34%) global DNA methylation, defined on mCyt median value, and the survival rate evaluated by Kaplan—Meier analysis. *RFC1 80AA* patients with low mCyt had a significantly worse survival in comparison both with GA and GG subjects, either with low or high mCyt levels (p = 0.002) (data not shown). Since the *RFC1 80G* carriers exhibited similar survival rate curves, the two groups were merged and the Kaplan—Meier analysis repeated. The plotted curves highlighted that *RFC1 80AA* patients with low mCyt levels had a poorer survival rate, as compared with G allele carriers (p<0.0001) (Fig 2). Noteworthy, all the five patients characterized by *RFC1 80AA* with low mCyt were deceased at the time of observation, whereas both the *RFC1 80G* carriers and *RFC1 80AA* with high mCyt showed better survival rates (71.1% and 50%, respectively). No statistically significant differences were found when comparing the *RFC1 80G* carriers and the *RFC1 80AA* with high mCyt (p = 0.919) and when comparing *RFC1 80AA* with low mCyt and *RFC180AA* with high mCyt (p = 0.209) (Fig 2).

### *RFC1 80G>A* genotypes and mortality risk according to global DNA methylation

**Fig 3:**
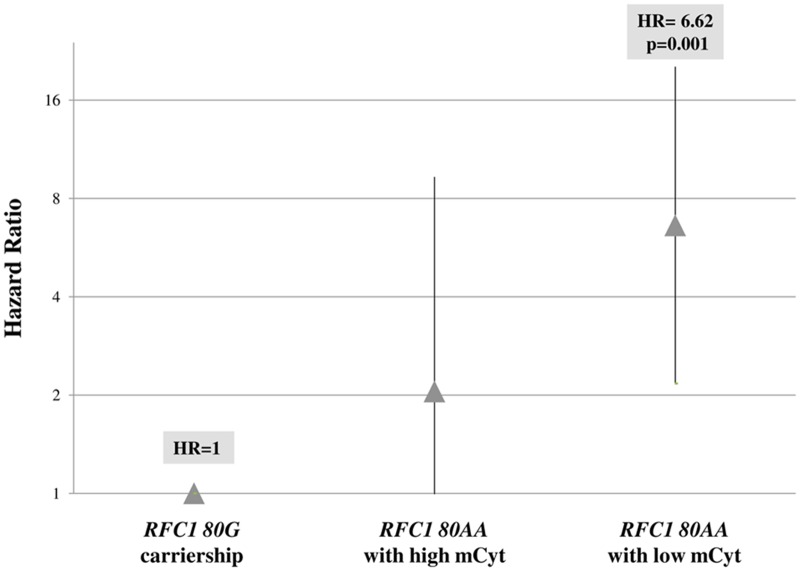
Mortality risk by Hazard Ratio for *RFC1 80AA* genotype with either high or low mCyt levels. The *RFC1 80AA* genotype with low mCyt levels (≤5.34%), had a higher Hazard Ratio (HR) as compared to *RFC1 80G* carriership (*RFC1 80GA* plus *RFC1 80GG*) (HR = 6.62, 95% CI 2.17–20.25, p = 0.001). The HR for *RFC1 80AA* genotype with high mCyt levels (>5.34%) did not differ from the *RFC1 80G* carriership group (HR = 2.05, 95% CI 0.45–9.32, p = 0.351).

The analysis of mortality risk was performed by setting the *RFC1 80G* carriership as the reference group with Hazard Ratio (HR) = 1 and the mortality risk associated to *RFC1 80AA* genotype with high or low mCyt levels was calculated. Subjects carrying the *RFC1 80AA* genotype and high mCyt had an HR = 2.05, not statistically different from the reference group, whereas the concomitant presence of *RFC1 80AA* genotype and low mCyt was associated to a 6.62-fold higher HR, as compared to *RFC1 80G* carriers (p = 0.001) (Fig 3).

**Fig 4:**
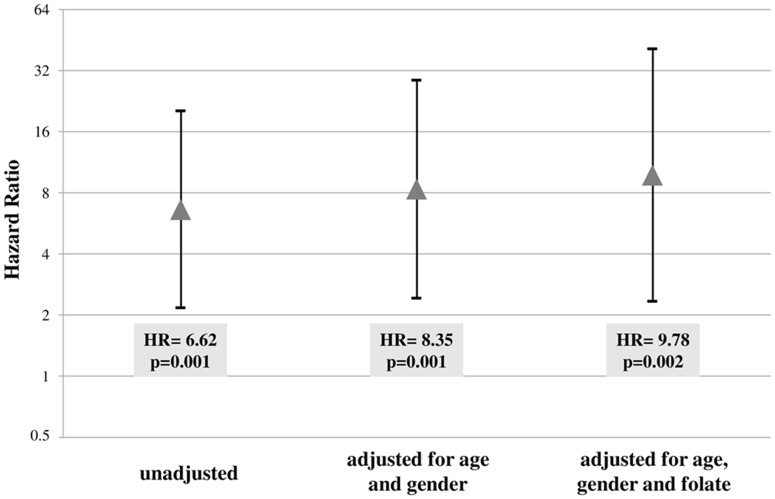
Mortality risk associated to carriership of the *RFC1 80AA* genotype with low mCyt using crude Hazard Ratio and after adjustments for age, gender and folate. The unadjusted Hazard Ratio (HR) was 6.62 (95% CI 2.17–20.25, p = 0.001) and the value significantly increased after adjustments for age and gender (HR = 8.35, 95% CI 2.42–28.67, p = 0.001) and for age, gender and plasma folate concentrations (HR = 9.78, 95% CI 2.34–40.94, p = 0.002).

The HR significantly increased when the analysis was performed after adjustments for age and gender (HR = 8.35 CI 2.42–28.67, p = 0.001) and for age, gender and plasma folate concentrations (HR = 9.78 CI 2.34–40.94, p = 0.002) (Fig 4).

## Discussion

The results of this study indicates that primary liver cancer patients carrying the *RFC1 80AA* genotype associated with low DNA methylation have a significantly poorer survival rate with a higher mortality risk, as compared with G allele carriers.

The Reduced Folate Carrier 1 (*RFC1*, official symbol *SLC19A1*) gene is located on chromosome 21 and encodes for an ubiquitously expressed transmembrane protein that serves as a bi-directional transporter of reduced folate species such as 5-methyl THF, the main circulating active form of folate [32, 33] and it is involved in both methylation pathway and nucleotides synthesis.

Chango et al., described a *RFC1* polymorphic variant (rs 1051266) consisting in the substitution of a guanine to an adenine (G>A) at position 80 of gene sequence, causing an arginine to histidine substitution at amino acid position 27 [29]. The exact functional relevance of this variant is, however, still unclear [34, 35]. In several human and animal studies no significant differences were observed both in plasma and red blood cell folate concentration in association with *RFC1 80G>A* genotypes [29, 36–42]. Results from the present study confirmed the lack of differences in plasma folate concentrations according to the *RFC1 80G>A* genotypes. The *RFC1 80G>A* polymorphism might be associated to a lower availability of methyl groups at tissue level for methylation reaction rather than to an evidently reduced level of plasma folate. The relationship among the *RFC1 80G>A* polymorphism, cancer risk and clinical outcome have been the objects of several studies, though with contrasting reports.

*RFC1 80G* allele was associated with an increased risk of head and neck carcinoma [43, 44], whereas an increased risk of gastric and esophageal cancer was found in association with the *RFC180AA* genotype [45]. The *RFC1 80AA* genotype was also associated with increased risk for acute lymphoid leukaemia with worse outcome [46], higher chance of relapse and poorer survival [47, 48] as compared to GA and GG genotypes. On the contrary, in patients affected by rectal cancer the *RFC1 80AA* genotype was associated with a more favourable prognosis [49]. On the other hand, no association was found between *RFC1 80G>A* genotypes and breast cancer risk [50] or survival [51].

Global DNA hypomethylation is associated with genomic instability and compromised gene repression in genomic regions that are usually silenced in normal cells [11, 12, 52]. The consequence of this event may entail the expression of proto-oncogenes as well as the activation of viral and parasitic transposons [52, 53]. Moreover, it has been consistently demonstrated that DNA hypomethylation is able to increase the immunogenicity and the immune recognition of cancer cells, thus affecting transcriptional deregulation and tumour aggressiveness [52]. Most of the studies showing an aberrant DNA methylation evaluated this epigenetic feature of DNA at the level of the specific tissue affected by the malignant process. One key point for discussion is, in fact, the finding of low methylation levels measured in PBMCs DNA. Considering that epigenetic mechanisms including methylation are usually considered tissue-specific, some speculations in this regard are needed. It has been suggested that a global DNA hypomethylation status in PBMCs might represent a potential epigenetic marker for cancer [13] and low levels of mCyt are usually associated to increased cancer risk and to poor prognosis [13–16]. In a previous study we demonstrated that patients affected by different types of cancer had a significantly lower global methylation in PBMCs DNA as compared to cancer-free controls and that DNA hypomethylation was also associated with a higher risk for cancer development [17]. Moreover, in a recent study in primary liver cancer patients we demonstrated that low mCyt levels in PBMCs DNA were related to a worse prognosis, suggesting a putative prognostic relevance of DNA methylation [18]. A recent prospective case-control study reported that *RFC1 80G>A* is associated with LINE-1 undermethylation as measured in peripheral blood DNA of women who developed breast cancer as compared with breast cancer-free controls [54]. Nevertheless, whilst the *RFC1 80G>A* variant was associated to lower methylation, no relationship was found between the presence of the variant and cancer risk [54].

The results of the present study demonstrate that the *RFC1 80AA* genotype is significantly associated with a poorer life expectancy in primary liver cancer patients. Moreover, when the mCyt levels were stratified as either high (>5.34%) or low (≤5.34%), patients with the *RFC1 80AA* genotype and low mCyt showed a significantly worse survival rate and a higher mortality risk as compared to the *RFC1 80G* carriers, even after adjustments for sex, age and folate concentrations. Noteworthy, the five patients characterized by the *RFC1 80AA* genotype and low mCyt levels were all deceased at the time of the observation.

The mechanism by which global DNA hypomethylation in the presence of the *RFC1 80AA* genotype may entail a poorer prognosis in patients affected by primary liver cancer remains to be explored. RFC1 is a key transmembrane bi-directional transporter of 5-methyl THF, the main circulating active form of folate, and it is involved in both methylation pathway and nucleotides synthesis. The present results showed that the AA genotype is associated to lower survival in primary liver cancer and that the AA genotype and concomitant low mCyt levels have a significantly poorer survival rate with a higher mortality risk. One hypothesis for the explanation of this finding is that RFC1 might be involved in carcinogenesis by affecting both methylation reactions and DNA repair capacity.

Even if the results of this study suggest that primary liver cancer patients showing *RFC1 80AA* genotype and low global DNA methylation at the enrolment, are at risk for poorer survival, further validation is required to assess whether this genetic-epigenetic fingerprint may be used as a clinically valuable prognostic tool. The reliable identification of prognostic molecular biomarkers may assume an important role in the clinical decision process for tailored therapeutic approaches in cancer treatment and in the improvement of cancer prognosis. Further investigations are certainly warranted to clarify the biological effect of *RFC1 80G>A* and the prognostic significance of PBMCs global DNA methylation assessment in primary liver cancers.

## Abbreviations

HR: Hazard Ratio
mCyt: methylcytosine
PBMCs: peripheral blood mononuclear cells
*RFC1*: Reduced Folate Carrier 1
